# Acrylamide‐Induced Developmental and Molecular Toxicity in *Drosophila melanogaster*: The Role of Vanillic Acid in CncC/Keap1 Modulation

**DOI:** 10.1002/jbt.71088

**Published:** 2026-08-26

**Authors:** Fahriye Zemheri‐Navruz, Sinan Ince, Gamze Ataman

**Affiliations:** ^1^ Department of Molecular Biology and Genetics Faculty of Science, Bartın University Bartın Türkiye; ^2^ Department of Pharmacology and Toxicology Faculty of Veterinary Medicine, Afyon Kocatepe University Afyonkarahisar Türkiye; ^3^ Cappadocia Vocational School Pathology Laboratory Techniques Program, Cappadocia University Nevşehir Türkiye

**Keywords:** acrylamide, *D. melanogaster*, gene expression, genotoxicity, vanillic acid

## Abstract

Acrylamide, a chemical used in industrial applications, has been classified as an environmental toxin owing to its widespread contamination of drinking water and groundwater resources. Vanillic acid (VA) is a phenolic compound naturally occurring in numerous dietary sources and medicinal plants. VA exhibits a wide range of pharmacological properties, including antioxidant, anti‐inflammatory, and neuroprotective activities. This study investigated the protective effects of VA against acrylamide‐induced oxidative stress in *Drosophila melanogaster* at developmental, biochemical, and molecular levels. *D. melanogaster* were exposed to acrylamide (2 mM) alone or with VA at three doses (0.1, 0.5, and 1 mg/L). Acrylamide exposure elevated reactive oxygen species and lipid peroxidation, decreased antioxidant enzyme activities (SOD, CAT) and glutathione (GSH) levels, increased DNA damage by the Comet assay, upregulated mRNA expression of stress‐related genes (*SOD*, *CAT*, *gclc* and *hsp70*) and detoxification‐pathway components (*Keap1* and *CncC/Nrf2*), and adversely affected developmental parameters (larval toxicity, crawling, climbing, survival, food intake, body weight and length). Co‐administration of VA dose‐dependently reduced lipid peroxidation and ameliorated antioxidant enzyme activities and DNA damage. VA also modulated the impaired developmental parameters of acrylamide‐exposed larvae. These findings indicate that VA possesses considerable protective potential against acrylamide toxicity, with important implications for environmental and human health.

## Summary

- Acrylamide induced oxidative stress and developmental toxicity in *Drosophila melanogaster*.
- Acrylamide upregulated *SOD, CAT, gclc, and hsp70* via the CncC/Keap1 pathway.
- VA dose‐dependently attenuated acrylamide‐induced lipid peroxidation and DNA breaks.
- VA restored AchE activity, mitigating the neurotoxic effects of acrylamide.
- First report of VA's ameliorative effect on acrylamide toxicity in *D. melanogaster*.

## Graphical abstract

Vanillic acid, a dietary phenolic, dose‐dependently protects *Drosophila melanogaster* from acrylamide‐induced oxidative damage, DNA strand breaks, and developmental toxicity by modulating CncC/Keap1 redox signaling—establishing vanillic acid as a candidate natural protective agent against a major environmental contaminant.

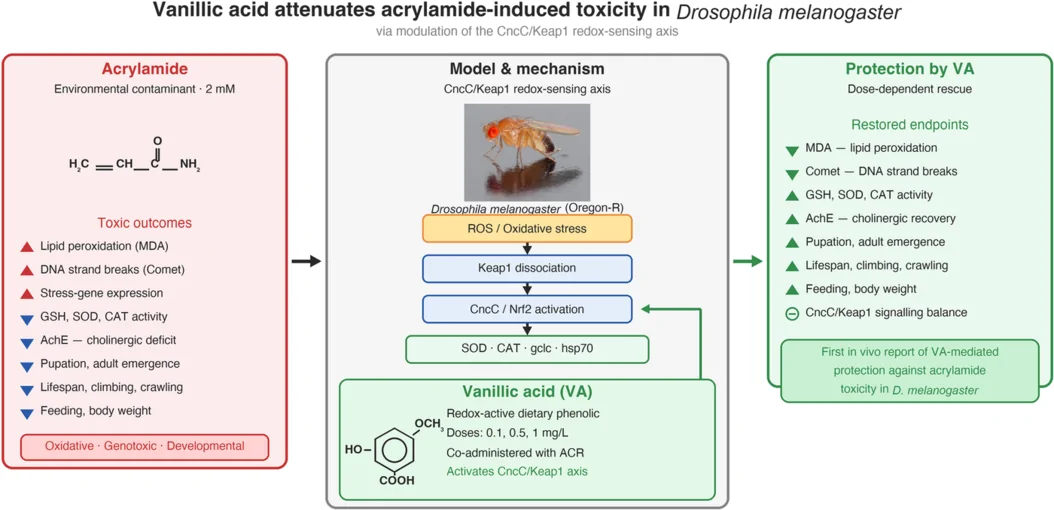

## 1 Introduction

Acrylamide (C_3_H_5_NO) is one of the most extensively investigated chemicals worldwide, owing to its polar structure that allows ready solubility in solvents such as water, alcohol, and ether. This compound, which can occur in monomeric or polymeric forms, is widely employed in a variety of industrial processes, including the paper, textile, cosmetics, and dye industries [1]. However, this widespread use has resulted in serious environmental contamination, particularly of drinking and groundwater resources, leading to the classification of acrylamide as an environmental toxin. In addition, acrylamide is readily absorbed through mucosal surfaces, the lungs, and the skin, posing a substantial occupational hazard to workers in the relevant industries [1, 2]. Following the 2002 report by Swedish scientists demonstrating the presence of acrylamide in thermally processed foods, major regulatory bodies including the U.S. Food and Drug Administration, the World Health Organization, and the European Food Safety Authority have acknowledged the need for more comprehensive investigation of this compound. Furthermore, the International Agency for Research on Cancer has classified acrylamide as a probable human carcinogen [3, 4].

Subsequent studies have confirmed that acrylamide is generated through the Maillard reaction during the heat treatment of foods such as meat, fried potatoes, and baked products. Particularly high concentrations of acrylamide have been observed in foods rich in amino acids such as asparagine and starch‐rich foods such as potatoes [5, 6]. Physiologically based toxicokinetic models have been developed to assess the tolerable daily intake levels of this compound. According to these assessments, daily acrylamide intake of 40 µg/kg has been associated with neurotoxicity, while a daily intake of 2.6 µg/kg has been suggested to be sufficient to elicit carcinogenic effects [7]. These findings have rendered acrylamide‐induced toxicity studies indispensable, and have underscored the need for more comprehensive investigations to fully elucidate the impact of this compound on living organisms, particularly in light of its presence in food products. To this end, scientists have examined acrylamide‐induced toxicity in various model organisms, and the accumulated evidence has revealed that acrylamide elicits multifaceted adverse outcomes including developmental delay, neurotoxicity, genotoxicity, reproductive toxicity, and carcinogenic effects [8, 9, 10].

Vanillic acid (VA), a derivative of benzoic acid, is widely used in the food industry as a flavoring agent, preservative, and food additive [11]. As a phenolic molecule, VA is also recognized as the oxidized form of vanillin [12]. This compound is naturally distributed across a wide variety of sources, including grains, herbs, fruits, green tea, several beverages, beers, and wines [13]. Studies in the literature have demonstrated that VA possesses a broad spectrum of pharmacological properties, including antioxidant, anti‐inflammatory, immunostimulant, neuroprotective, hepatoprotective, cardioprotective, and antiapoptotic activities. Moreover, this compound has been reported to exhibit anticancer, antiobesity, antidiabetic, antibacterial, and anti‐inflammatory effects [14]. Despite its considerable therapeutic potential and favorable safety profile, research on the use of VA as a nutraceutical or therapeutic agent is still ongoing, and further scientific investigation in this field is warranted.

Various studies conducted on model organisms have clearly demonstrated that acrylamide elicits considerable adverse effects in *Drosophila melanogaster* [15]. Acrylamide has been reported to cause behavioral, developmental, and reproductive abnormalities, to substantially alter numerous biochemical stress parameters, and to induce serious cellular damage in flies [16]. However, a thorough review of the literature reveals that no study has thus far investigated the mitigation and/or amelioration of these adverse effects of acrylamide via VA administration [17]. This important gap necessitates further investigation of the potential interaction between these two compounds and provides the basis for an original research question in the field. Accordingly, the present study was designed to address this scientific gap by evaluating whether co‐administration of VA can attenuate the deleterious health effects of acrylamide and whether VA may serve as a protective compound against other toxic agents such as acrylamide.

The findings of the present study aim to contribute to the scientific literature both by addressing the mitigation of acrylamide‐induced toxicity and by elucidating the protective effects of VA. Investigations conducted using the *D. melanogaster* model organism have allowed the biological interactions between acrylamide and VA to be examined at developmental, biochemical, and molecular levels. This multi‐dimensional approach affords a comprehensive understanding of the relationship between these two compounds and provides important data to guide future research. Moreover, given that VA is a natural compound with a broad spectrum of applications, the present findings hold value across multiple domains, including food safety, public health, and industrial applications [18]. In light of these considerations, the present study is expected to serve as a scientific reference for researchers seeking to develop natural protective strategies against widespread and hazardous toxic agents such as acrylamide.

## 2 Materials and Methods

### 2.1 Chemicals

Acrylamide (CAS No: 79‐06‐1) and VA (CAS No: 121‐34‐6) were obtained from Sigma (St. Louis, MO, USA), and all other analytical‐grade chemicals were purchased from commercial suppliers.

### 2.2 *D. melanogaster* Stock and Culture

The wild‐type, non‐mutant *D. melanogaster* Oregon‐R strain maintained at the Molecular Biology and Genetics Research Laboratory of the Faculty of Science, Bartın University, was used in this study. Flies were reared in transparent culture vials on a standard cornmeal–sugar–yeast–agar medium under controlled conditions of 24°C, 60%–70% relative humidity, and a 12/12 h light/dark photoperiod [19, 20]. All experiments involving *D. melanogaster* were conducted in accordance with the ARRIVE guidelines for the reporting of in vivo experiments.

### 2.3 Experimental Design

A total of nine experimental groups were established. In the treatments, 2 mM acrylamide [9, 21] dissolved in DMSO (0.1% v/v) and VA at 0.1, 0.5, and 1 mg/L [22] were administered alone or in combination.

### 2.4 Developmental Parameters

For larval toxicity testing, 50 third‐instar larvae were placed in each group; the numbers of pupae and emerging adults were recorded at the end of day 10 under conditions of 25°C and 60% humidity, and the experiment was performed in two replicates [23, 24].

For the larval crawling assay, third‐instar larvae collected according to the methods of Parimi et al. [25] and Dan et al. [26] were washed with ×1 PBS and placed on graph paper covered with 2% agarose; crawling distances over 1 min were video‐recorded, and the mean number of grid lines crossed by nine larvae per group was calculated across three replicates. For the climbing assay, 30 adult male flies per group were transferred to vials marked at 3 cm following 10 days of incubation; after being gently tapped to the bottom, individuals crossing the mark within 15 s were counted from video recordings, and the mean of three replicates was determined [26]. For the lifespan assay, 50 adult male flies were placed in each setup and monitored until all individuals died; the medium was renewed weekly, and dead flies were counted daily, and the experiment was performed in two replicates [26, 27]. For the larval food intake analysis, 30 third‐instar larvae were maintained for 24 h in groups containing 1% brilliant blue dye, following the methods of Tanimura et al. [28] and Senthil Kumar et al. [29]; samples were homogenized in phosphate buffer (50 mM, pH 7.4), centrifuged at 12,000*g* for 15 min, and the supernatant was read at 630 nm. For larval body weight and length measurements, 10 third‐instar larvae were selected from each group across three replicates following the method of Mu et al. [30]; after washing in PBS, larval weight was determined gravimetrically, and length was measured using millimetric paper.

### 2.5 Biochemical Analyses

For each group, 50 adult flies were collected at the end of Day 10 without distinction by sex [31]. For Drosophila homogenate supernatants were used for MDA, GSH, SOD, CAT, AchE, and protein measurements. All biochemical analyses were performed with six biological replicates (three biological replicates for AchE). For drosophila homogenization, 0.05 g of drosophila was mechanically and ultrasonically homogenized in 0.5 mL of cold 50 mM KH_2_PO_4_ (pH 7.0) and centrifuged at 5000 rpm for 15 min [32]. Malondialdehyde (MDA) analysis was performed according to the method of Ohkawa et al. [33]; the absorbance of the MDA–TBA complex was measured at 532 nm, and results were expressed as nmol/µg protein. Glutathione (GSH) analysis was performed according to the method of Beutler et al. [34]; supernatants were precipitated and filtered, then mixed with phosphate buffer and DTNB, and the absorbance was measured at 412 nm. Results were expressed as nmol/µg protein. Superoxide dismutase (SOD) activity was determined by the method of Sun et al. [35], based on the inhibition of NBT reduction to formazan, with absorbance read at 560 nm. Results were expressed as U/µg protein. Catalase (CAT) activity was determined according to the method of Sinha [36] at 570 nm and expressed as U/µg protein. Acetylcholinesterase (AchE) activity was determined using a competitive ELISA kit (LOT: WS180VN47920, Elabscience; Houston, TX, USA). Following the addition of avidin–HRP conjugate and TMB substrate, the reaction was terminated with stop solution, and the resulting absorbance was measured at 450 ± 2 nm; concentrations were calculated against a standard curve. Protein concentrations were determined using a Modified Lowry Protein Assay Kit (#SK404, Bio Basic Inc., Canada) at 750 nm. All spectrophotometric measurements were performed using a Thermo Scientific Multiskan GO Microplate Spectrophotometer (Thermo Fisher Scientific, USA).

### 2.6 Molecular Analyses

For the Comet assay, 30 adult flies per group were used, and the experiment was performed in three replicates. Slides were coated overnight with 1% NMA (normal melting‐point agarose); on the day of the experiment, 0.5% LMA (low‐melting‐point agarose) was melted in a microwave and kept at 40°C. Ten flies were disrupted on the slide in 100 µL of Hank's Balanced Salt Solution (HBSS), mixed with 100 µL LMA, transferred to NMA‐coated slides, covered with coverslips, and dried at 4°C. Slides were maintained in lysis solution for 1 h at 4°C and equilibrated for 15 min in electrophoresis buffer; alkaline electrophoresis was then performed at 24 V and 300 mA for 40 min. After neutralization in 0.4 M Tris buffer (pH 7.5) for 5 min and three rinses with distilled water, slides were stained with ethidium bromide and examined under a fluorescence microscope (Zeiss, Germany). Damage was scored on a 0 (no damage) to 4 (maximum damage) scale in 100 randomly selected cells.

For gene expression analysis, 50 adult flies per group were used, and analyses were performed in three biological replicates following 10 days of exposure. Total RNA was isolated from tissues stored at −80°C using the GeneJET RNA Purification Kit (Thermo Fisher Scientific, USA); RNA quality and quantity were determined at A260/A280 using a Multiskan FC Microplate Photometer (Thermo Fisher Scientific, USA). One microgram of RNA was incubated with 1 µL of ×10 reaction solution and 1 µL of RNase‐free DNase I (Thermo Fisher Scientific, USA) in a 10 µL volume for 30 min at 37°C and 10 min at 65°C. cDNA synthesis was performed using the RevertAid H Minus First Strand cDNA Synthesis Kit (Thermo Fisher Scientific, USA). Primers (Table 1) specific to *D. melanogaster* were designed using NCBI to target the housekeeping gene *rp49*, the antioxidant‐related genes *SOD*, *CAT*, and *gclc*, the heat shock protein gene *hsp70*, and the *Keap1* and *Nrf2 (CncC)* genes. Amplification was performed on a Bio‐Rad real‐time PCR system (Bio‐Rad, USA), and Ct values were evaluated using the $2^{‐\Delta \Delta C_{t}}$ method [37]; *rp49* served as the endogenous control, and analyses were completed using the Bio‐Rad CFX Manager 3.1 software. Primer annealing temperatures were optimized by gradient PCR. To ensure uniform template loading and minimize inter‐well variability, a common master mix containing equally distributed pooled cDNA was aliquoted first, with primers added last. All qPCR reactions were run in technical triplicate, and amplification specificity was confirmed by melt‐curve analysis, which yielded a single peak per primer pair consistent with the expected amplicon sizes (Table 1). Formal efficiency calibration by standard‐curve dilution was not performed and is acknowledged as a limitation.

**Table 1:** Primer sequences used in the study.

Gene	Primer sequence (5′ → 3′)	NCBI accession no.	Product (bp)
*Keap1*	F: GCACTCGCCCATGCAATATG	NM_169744.3	131
	R: GAATCGACCACCGCATGAGA		
*Nrf2 (CncC)*	F: GTTTTCAAGCTCACCACCAAT	NM_170053.3	138
	R: TTCGCTGCTTCTTCTTCCCT		
*SOD*	F: ACACGAGCTGAGCAAGTCAA	NM_057387.5	105
	R: CAGTGGCCGACATCGGAATA		
*CAT*	F: CAACCCCTTCGATGTCACCA	NM_080483.3	113
	R: TCTGCTCCACCTCAGCAAAG		
*gclc*	F: CGCAATACCAAAGTCGCGTT	NM_080266.3	197
	R: ACTCTGTTTCGCTCTCTGCC		
*hsp70*	F: GCCGACGAGGACGAGAAG	NM_169441.2	159
	R: CCGGATAGTGTCGTTGCACT		
*rp49 (RpL32)*	F: TCCAAGAAGCGCAAGGAGATT	NM_170461.3	159
	R: GAACTCGGCACTCGCACATC		

### 2.7 Statistical Analysis

All statistical evaluations were performed using GraphPad Prism 8.0. Normally distributed datasets were analyzed by one‐way ANOVA, and intergroup differences were determined using Tukey's multiple comparisons test. Results are presented as mean ± standard deviation, and *p* < 0.05 was considered statistically significant. To complement the *p* values, effect sizes (Cohen's d, with the Hedges' g small‐sample correction) and 95% confidence intervals for the principal biochemical, neurotoxicity, and genotoxicity comparisons were calculated and are provided in Table 2. In addition, pathway‐level Pearson correlation coefficients (r) were computed on group‐mean values across the nine experimental groups using Python 3 (pandas, scipy.stats); these correlations are summarized in Figure 6.

**Table 2:** Effect sizes and 95% confidence intervals (CI) for the principal biochemical, neurotoxicity, and genotoxicity endpoints.

Endpoint	Contrast	Δ (mean difference)	95% CI	Cohen's *d*	Hedges' *g*	*n*/group
MDA	Control → ACR	+3.09	[+3.00, +3.18]	+41.9	+38.6	6
GSH	Control → ACR	−13.10	[−13.48, −12.72]	−44.9	−41.5	6
SOD activity	Control → ACR	−11.01	[−11.46, −10.56]	−31.5	−29.0	6
CAT activity	Control → ACR	−5.20	[−5.28, −5.12]	−89.2	−82.3	6
AChE	Control → ACR	−2.02	[−2.10, −1.94]	−33.7	−31.1	6
Comet (DNA)	Control → ACR	+3.00	[+2.50, +3.50]	+13.6	+10.9	3
MDA	ACR → ACR + VA 1.0	−0.89	[−0.98, −0.80]	−12.1	−11.1	6
AChE	ACR → ACR + VA 1.0	+0.74	[+0.66, +0.82]	+12.3	+11.4	6
Comet (DNA)	ACR → ACR + VA 1.0	−1.83	[−2.32, −1.34]	−8.5	−6.8	3

*Note:* Mean differences (Δ) and effect sizes were derived from the group means ± SD reported in the Results (*n* = 6 biological replicates; *n* = 3 for the Comet assay). Cohen's *d* was computed as Δ/pooled SD; Hedges' *g* applies the small‐sample correction. A positive Δ denotes an increase in the second group relative to the first; 95% CIs were obtained as Δ ± *t* (0.975, df) × SE (df = 2*n*−2).

## 3 Results

### 3.1 Findings on Developmental Parameters

Acrylamide (ACR) exposure substantially suppressed pupation compared with the control group (control: 30.67 ± 0.58; ACR: 14.00 ± 2.00; *p* < 0.0001). With increasing doses of VA, pupal counts remained higher than those of the ACR group; significant recovery was observed particularly in the ACR + VA 0.5 (25.00 ± 1.00) and ACR + VA 1.0 (25.67 ± 1.53) groups. The number of female adults decreased with ACR (control: 16.33 ± 0.58; ACR: 4.00 ± 1.00; *p* < 0.0001), and a dose‐dependent increase was achieved with VA supplementation. A similar pattern was observed for male adult counts.

**Figure 1:**
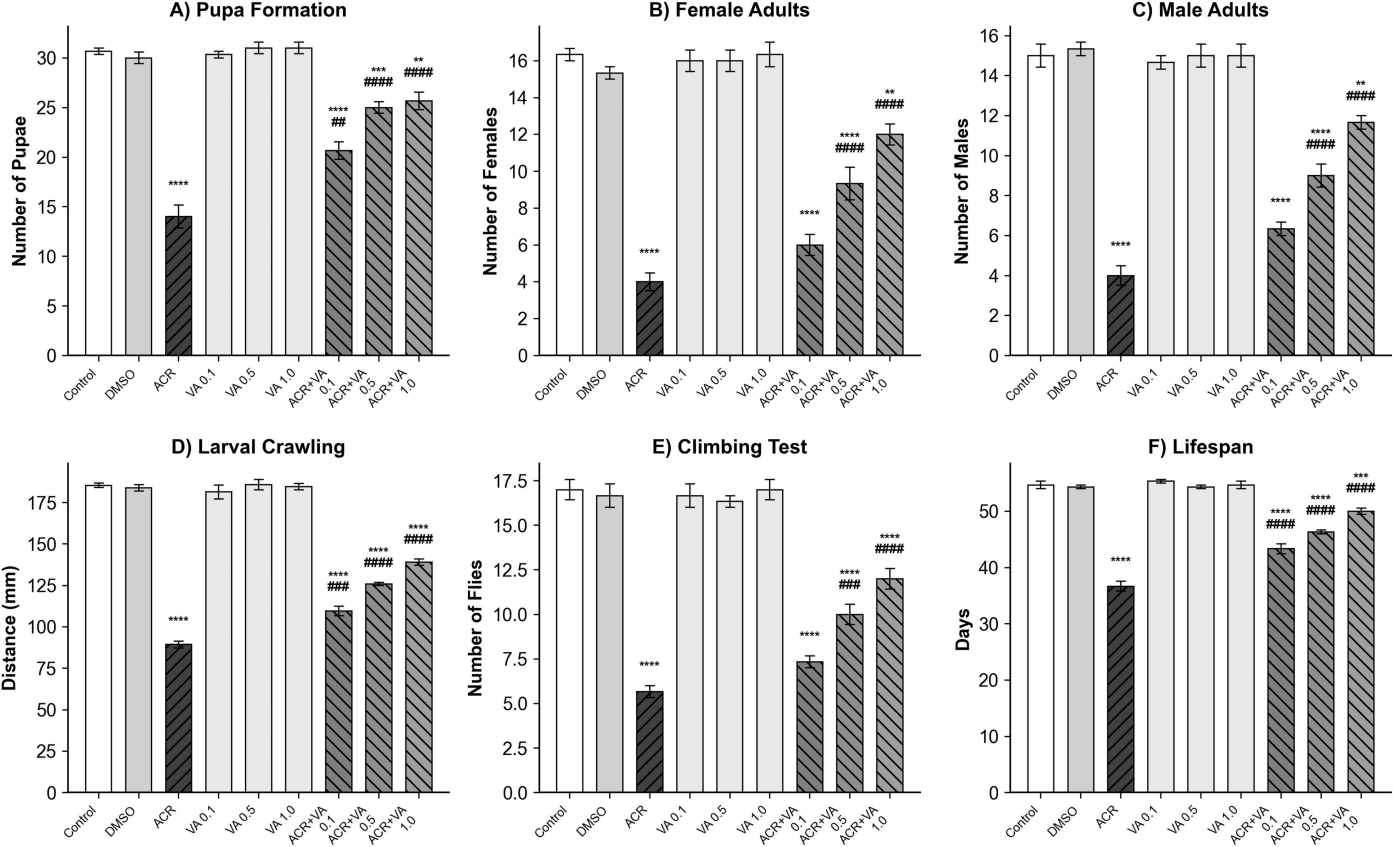
Effects of acrylamide (ACR, 2 mM) and vanillic acid (VA) at 0.1, 0.5, and 1 mg/L, alone and in combination, on developmental parameters: (A) pupation; (B) female adult counts; (C) male adult counts; (D) larval crawling distance; (E) climbing assay; (F) lifespan. Data are presented as mean ± SD. *Statistical significance versus control; #versus ACR group (*p* < 0.05).

Larval crawling distance was significantly reduced in the ACR group (control: 185.43 ± 2.40 mm; ACR: 89.33 ± 3.28 mm; *p* < 0.0001). VA at 0.1 (*p* = 0.0004), 0.5, and 1 mg/L (*p* < 0.0001) partially reversed this decline. In the climbing assay, ACR significantly suppressed motor performance in adult flies (control: 17.00 ± 1.00; ACR: 5.67 ± 0.58; *p* < 0.0001); whereas no significant improvement was observed at the 0.1 mg/L VA dose, the 0.5 (*p* = 0.0004) and 1 mg/L (*p* < 0.0001) doses significantly enhanced climbing capacity relative to the ACR group. Lifespan was shortened by ACR (control: 54.67 ± 1.15 days; ACR: 36.67 ± 1.53 days; *p* < 0.0001), and statistically significant prolongation relative to the ACR group was recorded at all VA doses (Figure 1). Furthermore, single‐treatment with VA at 0.1, 0.5, and 1 mg/L significantly increased larval food intake compared with the control (*p* < 0.0001).

### 3.2 Findings on Biochemical Parameters

**Figure 2:**
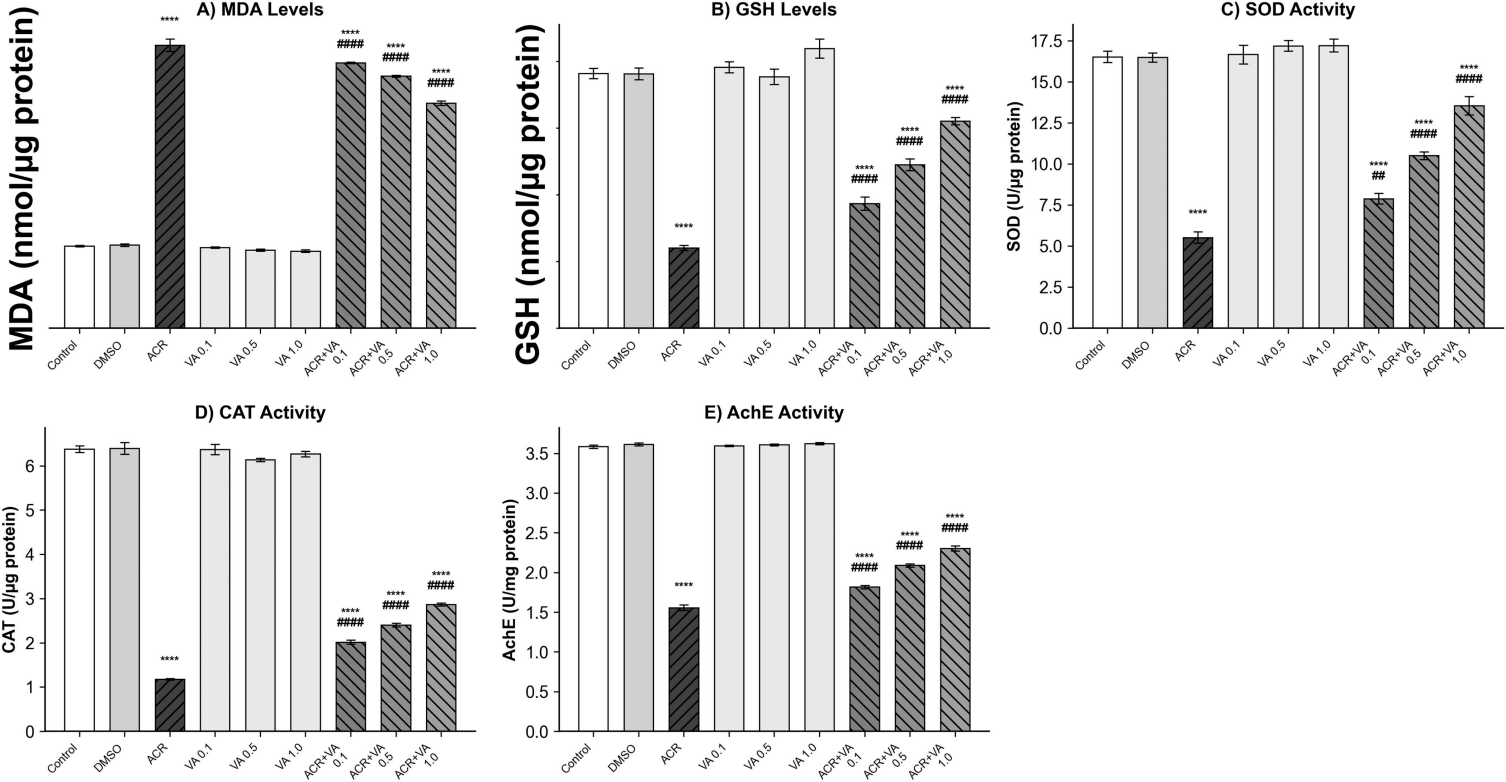
Effects of acrylamide (ACR, 2 mM) and vanillic acid (VA) at 0.1, 0.5, and 1 mg/L, alone and in combination, on biochemical parameters: (A) MDA levels; (B) GSH levels; (C) SOD activity; (D) CAT activity; (E) AchE activity. Data are presented as mean ± SD. *Statistical significance versus control; #versus ACR group (****p < 0.0001; ###*p* = 0.0001; ####*p* < 0.0001).

MDA levels showed a marked increase in the ACR group compared with the control (control: 1.26 ± 0.03; ACR: 4.35 ± 0.10; *p* < 0.0001). All three VA doses lowered MDA levels relative to ACR, with the most pronounced effect at 1 mg/L (ACR + VA 1.0: 3.46 ± 0.03; *p* < 0.0001). GSH content was reduced by ACR (control: 19.10 ± 0.38; ACR: 6.00 ± 0.16; *p* < 0.0001), and a concentration‐dependent recovery was recorded with VA. *SOD* activity decreased in the ACR group (control: 16.52 ± 0.35; ACR: 5.51 ± 0.35; *p* < 0.0001) and showed a gradual increase with VA supplementation. *CAT* activity exhibited a similar trend (control: 6.38 ± 0.08; ACR: 1.18 ± 0.02; *p* < 0.0001). AchE levels decreased with ACR (control: 3.58 ± 0.06; ACR: 1.56 ± 0.06; *p* < 0.0001) and showed a dose‐dependent increase with VA treatment (ACR + VA 0.1: 1.82 ± 0.04; ACR + VA 0.5: 2.09 ± 0.04; ACR + VA 1.0: 2.30 ± 0.06). DMSO and VA‐only groups did not deviate from control values (Figure 2).

### 3.3 DNA Damage and Feeding Findings

**Figure 3:**
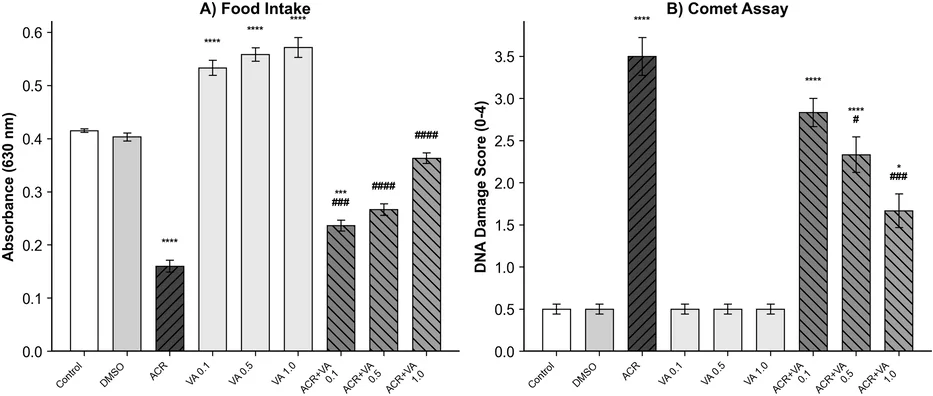
Effects of acrylamide (ACR, 2 mM) and vanillic acid (VA) at 0.1, 0.5, and 1 mg/L, alone and in combination: (A) larval food intake; (B) DNA damage levels (Comet assay). Data are presented as mean ± SD. *Statistical significance versus control; #versus ACR group (****p < 0.0001; *p = 0.0106; #*p* = 0.0106; ###*p* < 0.0001).

Comet scores demonstrated that ACR significantly increased DNA strand breaks in *D. melanogaster* (control: 0.50 ± 0.22; ACR: 3.50 ± 0.22; *p* < 0.0001). Damage scores in the ACR + VA 0.5 (2.33 ± 0.21) and ACR + VA 1.0 (1.67 ± 0.21) groups were significantly reduced compared with ACR. Feeding absorbance was decreased by ACR (control: 0.42 ± 0.00; ACR: 0.16 ± 0.01; *p* < 0.0001), with partial improvement at VA doses of 0.5 and 1 mg/L (Figure 3).

### 3.4 Changes in Gene Expression Levels

**Figure 4:**
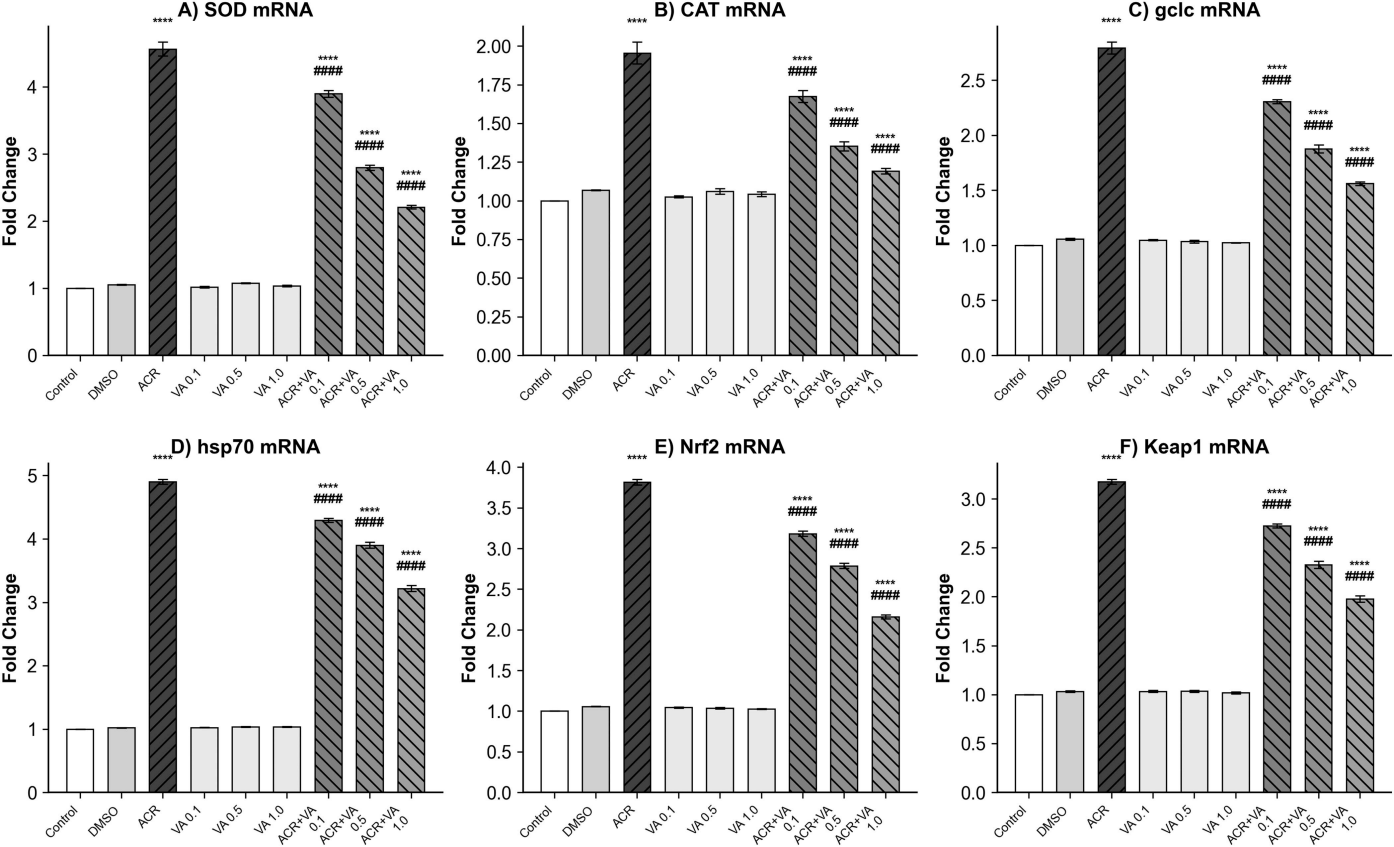
Effects of acrylamide (ACR, 2 mM) and vanillic acid (VA) at 0.1, 0.5, and 1 mg/L, alone and in combination, on gene expression levels (qRT‐PCR): (A) SOD; (B) CAT; (C) gclc; (D) hsp70; (E) Nrf2; (F) Keap1. Data are presented as fold change calculated using the $2^{-\Delta \Delta C_{t}}$ method. *Statistical significance versus control; #versus ACR group (****p < 0.0001; **p = 0.0065; ####*p* < 0.0001).

ACR exposure significantly upregulated the mRNA levels of all target genes investigated relative to control (*p* < 0.0001): *SOD* (4.57 ± 0.22‐fold), *CAT* (1.96 ± 0.07‐fold), *gclc* (2.79 ± 0.05‐fold), *hsp70* (4.90 ± 0.04‐fold), *Nrf2* (3.82 ± 0.04‐fold), and *Keap1* (3.18 ± 0.02‐fold). Co‐administration of the three doses of VA significantly reduced the expression of these genes relative to the ACR group (*p* < 0.0001). At the 1 mg/L VA dose, *SOD* (2.21 ± 0.05‐fold) and *Nrf2* (2.16 ± 0.02‐fold) showed the most prominent decrease. The gene expression profiles of the VA‐only and DMSO groups did not differ significantly from those of the control group (Figure 4).

### 3.5 Larval Morphological Parameters

**Figure 5:**
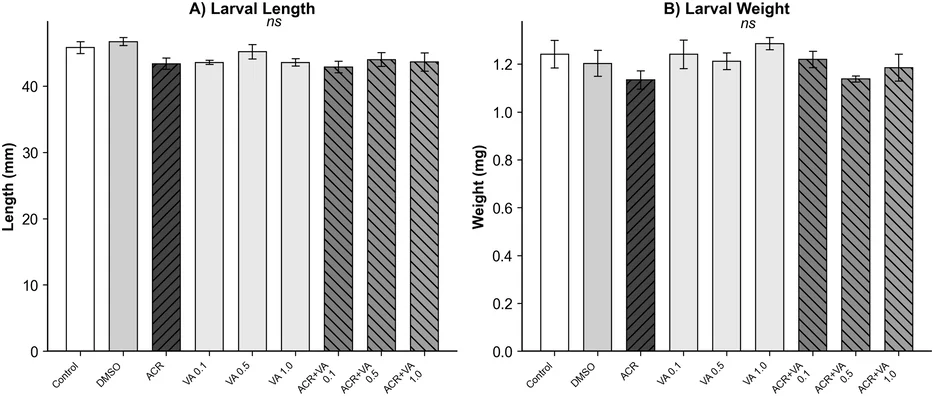
Effects of acrylamide (ACR; 2 mM) and vanillic acid (VA) at 0.1, 0.5, and 1 mg/L, alone and in combination, on larval morphological parameters: (A) larval length; (B) larval weight. Data are presented as mean ± SD. No statistically significant differences were detected between groups (ns).

No significant differences were detected between groups in larval length or weight (Figure 5).

All effect sizes greatly exceed the conventional threshold for a large effect (| *d* | ≥ 0.8), and the narrow 95% CIs indicate high precision, reflecting the low within‐group variability of the dataset.

## 4 Discussion

Acrylamide is one of the priority items on the global food safety agenda due to its inevitable presence in heat‐treated foods and the associated risk of chronic low‐dose exposure [38]. The data obtained in the present study indicate that acrylamide exerts multifaceted toxic effects in *D. melanogaster* at developmental, biochemical, and molecular levels, and that VA mitigates these effects in a dose‐dependent manner.

Although acrylamide‐induced developmental toxicity has been widely investigated in model organisms, to the best of our knowledge, only a limited number of studies have been conducted in the Drosophila model. Studies focusing on Drosophila development in the context of acrylamide toxicity have largely concentrated on two main areas, namely genotoxicity and reproductive toxicity. Within these areas, the sex‐linked recessive lethal (SLRL) test has demonstrated mutagenic effects of acrylamide in the germ line; this assay revealed reduced fertility across all offspring, several cases of sterility, and an overall increase in mortality. Wing‐spot tests have also confirmed the mutagenicity of acrylamide in somatic cells, with single and twin spots developing on the wings of adults derived from acrylamide‐exposed larvae [39]; Senthil [29]. One study reported that dietary exposure of *D. melanogaster* (Oregon K strain) to acrylamide resulted in concentration‐ and time‐dependent mortality, with surviving flies displaying significant locomotor deficits attributable to oxidative‐stress‐induced neuronal damage. Drosophila embryos were also reported to display signs of developmental toxicity, including alterations in the migration of border and cluster cells during developmental stages [9]. In another study, *D. melanogaster* exposed to high concentrations of acrylamide (25, 50, and 100 mg/kg) for 7 days exhibited markedly reduced survival, offspring emergence, activity, sleep, and locomotor behavior, indicating a clear loss of viability [40].

In a study examining the duration of larval and pupal stages and survival times in housefly (*Musca domestica*) larvae exposed to 82, 164, or 246 µg/g acrylamide, the survival times of larvae exposed to 82 and 164 µg/g were reduced by 50% and 85%, respectively, while the 246 µg/g concentration was lethal. In both groups, larval and pupal stages were prolonged by approximately 1.5 days compared with the control, with increased numbers of prohemocytes and intermediate cells, and decreased plasmatocyte size and number as well as granulocyte counts [41]. *D. melanogaster* larvae, in which the effects of acrylamide consumption and strain type on crawling performance were analyzed, only the strain factor was reported to exert a significant effect on the crawling‐speed parameter when acrylamide was administered at 24 and 48 mg/kg over a single day, whereas neither acrylamide consumption nor interaction effects were significant [42]. On the other hand, administration of 2 mM acrylamide to *D. melanogaster* over a 5‐day period was reported to shorten lifespan and to diminish larval crawling and adult climbing behaviors [43]. It has also been reported that the adverse effects of acrylamide on developmental parameters in *D. melanogaster* can be attenuated by low‐molecular‐weight chitosan and pharmacologically active substances such as curcumin and thymoquinone [9, 43]. Consistent with these reports, in the present study, VA administration in *D. melanogaster* (particularly at 0.5 and 1 mg/L) was found to attenuate the larval toxicity induced by acrylamide, while increasing larval crawling distance, climbing distance, lifespan, and food intake, all of which had been reduced by acrylamide. This indicates a beneficial effect of VA on developmental parameters. Osorio‐Paz et al. [44] similarly reported that VA significantly extended lifespan in *C. elegans* by enhancing stress resistance, lending support to the dose‐dependent protective profile observed in our study.

The most well‐established mechanism through which acrylamide exerts toxicity in Drosophila, laboratory animals, and humans is the induction of oxidative stress through enhanced production of reactive oxygen species [15, 45]. Consistent with this, several biochemical parameters were altered in flies exposed to acrylamide in the present study. Increased lipid peroxidation was accompanied by decreased GSH content and reduced SOD and CAT activities; this likely results from the depletion of GSH—which contributes to acrylamide detoxification—together with diminished activity of antioxidant enzymes that counter oxidative stress. These findings are consistent with previous reports in Drosophila and rodent models, which have established GSH conjugation as a major detoxification pathway for acrylamide [15, 46, 47]. Conversely, numerous studies have reported beneficial effects of various phytochemicals against acrylamide‐induced toxicity [45]. Recent studies have shown that the toxic effects of acrylamide can be reversed by antioxidants such as curcumin, quercetin, and thymoquinone, all of which are recognized for their antioxidant properties [9, 48, 49]. Similarly, in the present study, VA was observed to alleviate acrylamide‐induced oxidative stress, thereby restoring biochemical homeostasis, owing to its potent pharmacological properties. Vinothiya and Ashokkumar [50] likewise reported that VA possesses both direct free‐radical scavenging capacity and the ability to enhance endogenous antioxidant systems.

The activity of AChE—an essential enzyme that can be employed as a neurotoxic marker in fly models—was also examined. AChE levels were significantly lower in acrylamide‐treated flies than in the other groups, whereas this level was found to be elevated in VA‐treated flies. Several studies have demonstrated that acrylamide exposure adversely affects key parameters such as AChE levels alongside oxidative‐stress markers [43]. The neurotoxicity of acrylamide is characterized by its destructive effects on peripheral nerve terminals and synaptic transmission, and changes in AChE activity represent an important indicator of this process. The partial restoration of cholinergic neurotransmission by VA constitutes a critical finding supporting the neuroprotective potential of this compound. The experimental study by Ahmadi et al. [51] reported that VA reduced memory impairment and oxidative stress induced by Aβ1‐40 exposure; furthermore, Ghaderi et al. [52] provided a detailed examination of the neuroprotective mechanisms underlying cognitive decline associated with diabetes in rats.

Acrylamide and its epoxide metabolite, glycidamide (GA), bind to hemoglobin or DNA to form adducts and interact with other proteins at the cellular level, eliciting cytotoxic and genotoxic effects [53]. Ansar et al. [54] reported that DNA damage was induced in rats exposed to acrylamide and that this damage was reduced by quercetin treatment. Another study highlighted that 2.5 µg/mL curcumin significantly reduced acrylamide‐induced DNA fragmentation, micronucleus formation, and cytotoxicity in HepG2 cells [55]. Olive oil and its lipophilic and hydrophilic fractions were reported to confer protection against DNA damage in rats with acrylamide‐induced cardiotoxicity [56]. Similarly, in the present study, acrylamide induced DNA damage in *D. melanogaster*, and VA exhibited a damage‐reducing effect attributable to its antioxidant properties.

In our study, acrylamide markedly upregulated the mRNA levels of SOD, CAT, gclc, and hsp70, indicating activation of the cellular antioxidant defense. However, this transcriptional induction did not translate into enzymatic activity, as SOD and CAT activities were concomitantly reduced—a discrepancy commonly attributed to direct enzyme inactivation and/or rapid consumption under sustained oxidative load. This dissociation between transcript and enzymatic activity is a well‐documented consequence of severe oxidative stress: reactive oxygen species and reactive carbonyl species can directly inactivate antioxidant enzymes through post‐translational modifications—including protein carbonylation, cysteine‐thiol oxidation, and adduct formation by reactive lipid‐peroxidation products—thereby lowering catalytic activity despite the compensatory upregulation of their transcripts [57, 58]. The molecular mechanisms underlying the stress response of *D. melanogaster*, which involve the CncC pathway, are functionally homologous to those of the mammalian Nrf2 pathway [59]. Under stress‐free conditions, CncC is sequestered in the cytoplasm by Keap1 [60, 61]. Upon exposure to electrophiles or ROS, the CncC–Keap1 interaction is disrupted, CncC accumulates in the nucleus, where it forms a heterodimer with Maf‐S, binds to AREs, and activates the transcription of target genes [62]. CncC regulates target genes resembling those of mammalian Nrf2 by encoding detoxification‐related enzymes and antioxidant proteins, including cytochrome P450 (CYP), GST, CAT, SOD, and GSH peroxidase (GPx) [63]. Furthermore, similarly to mammals, Drosophila CncC modulates protein homeostasis through the transcriptional regulation of 20S proteasomes and heat shock proteins (HSPs), thereby enhancing tolerance to various toxic conditions and strengthening resistance to oxidative stress [64, 65]. To date, no study has reported the effects of acrylamide on this pathway in *D. melanogaster*. The present study revealed that acrylamide exposure induced excessive activation of Nrf2 and concomitantly upregulated the expression of Keap1, SOD, CAT, gclc, and hsp70. On the other hand, the ameliorative effects of VA in diabetic nephropathic rats have been attributed to its potent free‐radical scavenging properties, downregulation of NF‐κB, TNF‐α, and COX‐2 proteins, and upregulation of Nrf‐2 in renal tissue [66]. In rats with pentylenetetrazole‐induced neurological damage, VA administration has been reported to substantially improve oxidative‐stress markers and to elicit a marked reduction in pentylenetetrazole‐induced seizures, accompanied by downregulation of GFAP and upregulation of Nrf2, HO‐1, and IGF‐1 in the CA3 hippocampal region; thus, VA was shown to exert neuroprotective and anti‐epileptic effects against epilepsy via its antioxidant activity through the Nrf2/HO‐1 pathway and IGF‐1 upregulation [67]. Consistent with these reports, the present study established that VA attenuates acrylamide‐induced oxidative stress through the Nrf2/Keap1 pathway. This apparent divergence reflects the context‐dependent nature of Nrf2 regulation. In the present model, acrylamide was the primary driver of CncC/Nrf2 activation; by acting as a direct free‐radical scavenger, VA lowered the acrylamide‐induced ROS burden and thereby reduced the demand for CncC/Keap1 activation, so that Nrf2 and its target transcripts returned toward baseline. In contrast, in mammalian disease models (e.g., diabetic nephropathy, pentylenetetrazole‐induced injury), VA can act as a hormetic inducer that upregulates Nrf2. Thus, the direction of Nrf2 modulation by VA is governed by the prevailing oxidative‐stress context rather than representing a contradictory effect.

From a translational perspective, the relevance of these findings is reinforced by the strong evolutionary conservation between *Drosophila* and mammals: the CncC/Keap1 axis is functionally orthologous to the mammalian Nrf2/Keap1 system, and the fly retains close homologs of approximately 75% of human disease‐related genes [68, 69]. Combined with its short life cycle, genetic tractability, and suitability for rapid in vivo screening, this positions *D. melanogaster* as a valuable first‐tier model for prioritizing protective phytochemicals such as VA. Nevertheless, quantitative extrapolation of protective doses and efficacy to mammals and humans will require confirmatory studies in vertebrate systems, since differences in absorption, metabolism, and tissue‐specific redox regulation may modulate the response.

**Figure 6:**
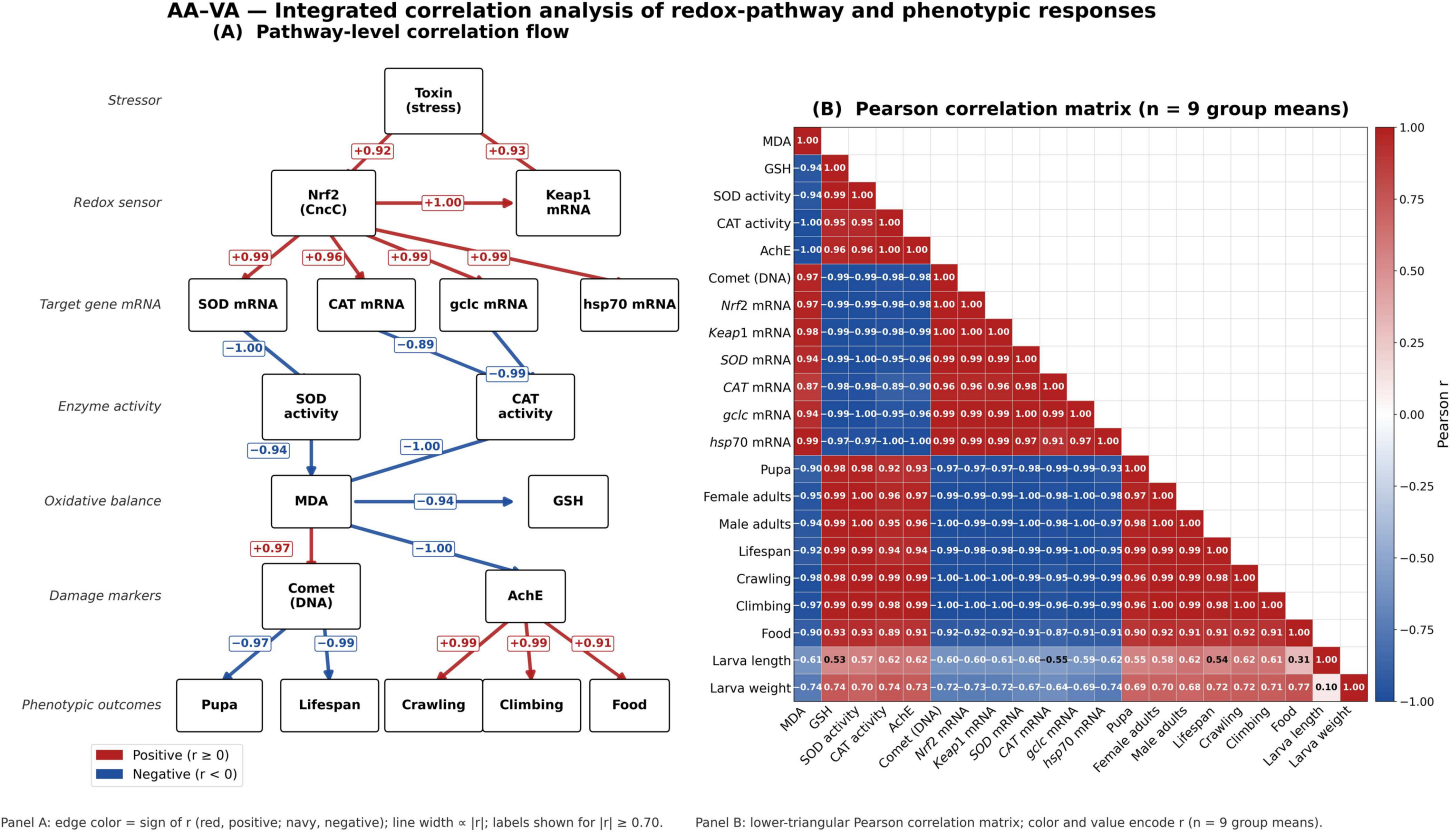
Integrated correlation analysis of redox‐pathway and phenotypic responses to acrylamide (ACR) and vanillic acid (VA) co‐treatment in *D. melanogaster*. (A) Pathway‐level correlation flow. Nodes represent the measured parameters arranged hierarchically across seven biological tiers, from stressor to phenotypic outcome. Edges depict Pearson correlation coefficients (r) calculated from group‐mean values across the nine experimental groups; the color of each edge encodes the sign of r (red, positive; navy, negative), the line width is proportional to |*r* |, and numerical labels are displayed for |*r* | ≥ 0.70. (B) Lower‐triangular Pearson correlation matrix covering all 21 measured parameters. The cell color gradient corresponds to Pearson *r* (red = positive, blue = negative); cell annotations indicate the r value; *n* = 9 group means.

Collectively, the integrated pathway‐level correlation analysis (Figure 6) confirmed and visualized this compensatory architecture: ACR‐induced upregulation of *CncC* and *Keap1* mRNAs was tightly coupled to all four target gene transcripts (*r* > +0.96, *p* < 0.001), whereas the transition from target‐gene mRNA to enzyme activity for *SOD* and *CAT* was inversely correlated (*r* = −1.00 and *r* = −0.89, respectively), supporting an adaptive transcriptional response that fails to translate into proportional enzymatic capacity under sustained oxidative load. Reduced enzyme activity in turn predicted higher MDA (*r* ≤ −0.94) and DNA strand breaks (*r* = +0.97), and ultimately shorter lifespan and impaired locomotion, providing a quantitative mechanistic chain from the redox sensor down to phenotypic outcome.

Several limitations should be considered when interpreting these findings. First, our analyses were conducted exclusively in *D. melanogaster*; although this model harbors approximately 75% of human disease‐related genes and is well‐validated in toxicological screening, extrapolation of the protective effects of VA to mammalian and human contexts requires further validation in vertebrate models. Second, although coordinated mRNA‐level changes were documented in CncC, Keap1, and downstream antioxidant genes, protein‐level confirmation (e.g., Western blot for Nrf2 nuclear translocation) and direct pathway perturbation (e.g., cnc/keap1 knockdown or pharmacological inhibition) were beyond the scope of this work and remain to be addressed in future studies. Accordingly, the CncC/Keap1 modulation reported here should be interpreted as transcriptional‐level evidence; protein‐level validation—including Western blotting or immunofluorescence for CncC/Nrf2 nuclear translocation—will be required to firmly consolidate the proposed mechanism. Third, the integrated correlation analysis (Figure 6) was performed on group‐mean values from the nine experimental conditions (six biological replicates per group for biochemistry; three for gene expression; two to three for behavioral and developmental endpoints, as detailed in Methods); while informative for visualizing pathway architecture, this approach has limited statistical power and would benefit from individual‐level multivariate validation. Finally, the present design tested a single sub‐lethal acrylamide dose with three VA concentrations under a fixed exposure regimen; consequently, a full acrylamide dose–response relationship was not established, and the efficacy of VA across acute versus chronic exposure durations was not assessed; chronic low‐dose exposures, sex‐specific responses, and the pharmacokinetic profile of VA in invertebrate systems remain to be elucidated. In addition, the present design did not include a reference antioxidant (e.g., N‐acetylcysteine, curcumin, or quercetin) as a positive control; although the protective profile of VA is interpreted in the context of these well‐characterized agents, direct head‐to‐head benchmarking in future studies would allow a more quantitative assessment of its relative efficacy.

## 5 Conclusion

The present study demonstrated that acrylamide—an important food‐borne toxin—induces oxidative stress, genotoxicity, neurotoxicity, and developmental toxicity in *D. melanogaster*, and that VA supplementation mitigates these adverse effects in a concentration‐dependent manner. VA reduced lipid peroxidation; improved GSH, SOD, CAT, and AchE levels; preserved DNA integrity; and modulated the cellular stress response via the CncC/Keap1 signaling pathway. The improvements observed in developmental parameters (pupation, crawling, climbing, lifespan, and feeding) corroborate the systemic protective profile of VA. The antioxidant and anti‐inflammatory properties of VA, together with its accessibility and favorable safety profile, provide a preliminary basis for considering this compound as a candidate protective agent, although the present evidence derives exclusively from an invertebrate model. The present findings indicate that VA represents a promising natural protective compound against food‐borne toxins such as acrylamide, contributing to food‐safety strategies. However, these findings should be regarded as proof‐of‐concept in *D. melanogaster*, and their therapeutic relevance to mammals and humans remains to be established through validation in vertebrate models. Future investigations are recommended to comprehensively examine the translational potential of VA for human health and ecosystem protection through chronic low‐dose exposure scenarios, alternative model organisms, and validation in mammalian systems.

## Author Contributions

**Fahriye Zemheri‐Navruz:** conceptualization, investigation, methodology, software, data curation, writing – review and editing, writing – original draft, project administration. **Sinan Ince:** methodology, validation, writing – review and editing, supervision, funding acquisition. **Gamze Ataman:** investigation, data curation.

## Conflicts of Interest

The authors declare no conflicts of interest.

## Data Availability

The data that support the findings of this study are available from the corresponding author upon reasonable request.
